# Microfluidic Tumor-on-a-Chip Model to Study Tumor Metabolic Vulnerability

**DOI:** 10.3390/ijms21239075

**Published:** 2020-11-28

**Authors:** Jose M Ayuso, Shujah Rehman, Mehtab Farooqui, María Virumbrales-Muñoz, Vijayasaradhi Setaluri, Melissa C Skala, David J Beebe

**Affiliations:** 1 Department of Pathology & Laboratory Medicine, University of Wisconsin, Madison, WI 53706, USA; mfarooqui@wisc.edu (M.F.); virumbralesm@wisc.edu (M.V.-M.); 2 Morgridge Institute for Research, 330 N Orchard Street, Madison, WI 53715, USA; shujah.rehman@wisc.edu (S.R.); mcskala@wisc.edu (M.C.S.); 3 Department of Biomedical Engineering, University of Wisconsin, Madison, WI 53706, USA; 4 The University of Wisconsin Carbone Cancer Center, University of Wisconsin, Madison, WI 53706, USA; 5 Department of Dermatology, University of Wisconsin-Madison, Madison, WI 53706, USA; vsetaluri@dermatology.wisc.edu

**Keywords:** tumor metabolism, tumor-on-a-chip, microfluidics, redox ratio

## Abstract

Tumor-specific metabolic adaptations offer an interesting therapeutic opportunity to selectively destroy cancer cells. However, solid tumors also present gradients of nutrients and waste products across the tumor mass, forcing tumor cells to adapt their metabolism depending on nutrient availability in the surrounding microenvironment. Thus, solid tumors display a heterogenous metabolic phenotype across the tumor mass, which complicates the design of effective therapies that target all the tumor populations present. In this work, we used a microfluidic device to study tumor metabolic vulnerability to several metabolic inhibitors. The microdevice included a central chamber to culture tumor cells in a three-dimensional (3D) matrix, and a lumen in one of the chamber flanks. This design created an asymmetric nutrient distribution across the central chamber, generating gradients of cell viability. The results revealed that tumor cells located in a nutrient-enriched environment showed low to no sensitivity to metabolic inhibitors targeting glycolysis, fatty acid oxidation, or oxidative phosphorylation. Conversely, when cell density inside of the model was increased, compromising nutrient supply, the addition of these metabolic inhibitors disrupted cellular redox balance and led to tumor cell death.

## 1. Introduction

One of the hallmarks of tumor cells is their constant and uncontrolled proliferation [1]. Consequently, tumor cells have a higher demand for macromolecules such as polysaccharides, lipids, or proteins, which imposes extensive metabolic rewiring [2]. Compounds targeting metabolic pathways associated with macromolecule synthesis (e.g., glycolysis, DNA, and amino acid synthesis) present a promising opportunity [3,4]. Tumor cells commonly rely on glycolysis to generate macromolecule precursors for other anabolic pathways (e.g., protein, lipid, and nucleic acid synthesis). Glycolysis also provides energy through oxidative phosphorylation and the tricarboxylic (TCA) cycle in the mitochondria, highlighting the importance of this pathway in cancer progression [2]. Thus, multiple compounds targeting critical glycolysis steps (e.g., 2-Deoxy-D-glucose, metformin hydrochloride) have been explored as cancer therapeutics [5,6]. On the other hand, fatty acids and proteins also provide an alternative energy source through the TCA cycle, supporting tumor cell survival [7]. Fatty acids enter the mitochondria and the TCA cycle in the form of acyl-CoA using the carnitine palmitoyltransferase (CPT) shuttle, producing adenosine triphosphate (ATP) through oxidative phosphorylation [8]. Hence, compounds targeting fatty acid oxidation (FAO), such as the CPT inhibitor etomoxir, have shown therapeutic potential [9]. Finally, inhibitors such as oligomycin, which target critical steps of the oxidative phosphorylation (i.e., ATP synthetase inhibitor), have also been explored as potential cancer therapeutics [3]. However, targeting one metabolic pathway may not suffice to block tumor cell metabolism since tumor cells can adapt their metabolism depending on the surrounding environment [4]. Therefore, blocking one metabolic pathway commonly leads to compensatory upregulation of alternative metabolic routes, allowing tumor cell survival [10]. Further, the limited supply of oxygen and nutrients in highly metabolically active tumors commonly leads to nutrient starvation, hypoxia, and waste product accumulation [11,12]. Therefore, tumor cells rewire their metabolic phenotype, and reduce cell proliferation and macromolecule demand [13,14]. Thus, solid tumors exhibit an evolving microenvironment where molecular (e.g., mutations) and environmental factors (e.g., nutrient availability) determine tumor metabolism. These observations contribute to the poor efficacy exhibited by these metabolic inhibitors in the clinic, highlighting the need for metabolic inhibitors capable of targeting the vast metabolic heterogeneity observed in vivo [15]. However, capturing this complex and spatially evolving tumor microenvironment with most Petri dish-based traditional models remains challenging.

Advances in microfabrication technologies have led to the development of advanced bioengineered in vitro models capable of capturing multiple features of human organ physiology [16,17]. There have been numerous studies demonstrating the potential of microfluidic devices for monitoring cell interactions in tumor development. Microfluidic devices provide excellent cell patterning control, allowing the generation of highly organized cultures. These properties allowed researchers to juxtapose endothelial cells and three-dimensional (3D) hydrogels to study tumor-induced angiogenesis. This approach led to the generation of vascular networks in vitro and highlighting the pro-angiogenic role of tumor cells. Enabled by this technology, researchers also used these vascular networks to study multiple steps of the metastatic cascade, such as tumor cell intravasation in the primary tumor and extravasation at the tumor site. Similarly, the combination of blood vessel models, 3D hydrogels, multicellular spheroids, and immune cells, allowed the study of complex tumor–immune interactions. These models analyzed immune recruitment, migration, penetration, and tumor killing, providing a versatile tool to evaluate the efficacy of breast cancer immunotherapies based on immune cell transfer and immune checkpoint inhibitors. Regarding tumor metabolism, previous microfluidic devices have been designed to generate oxygen, nutrient, pH, growth factor, and cell viability gradients, mimicking the organization observed in tumors in vivo [18,19,20,21,22]. However, most microfluidic devices designed to generate gradients relied on the use of external equipment to perfuse different solutions through parallel channels (e.g., 0% oxygen vs. 20% oxygen) to generate a gradient by passive diffusion, which imposes some technical hurdles for traditional cell biology labs. Thus, we used a microfluidic tumor-on-a-chip model that included tumor cells embedded in a 3D collagen matrix and a lumen located on one side of the microdevice [18]. This lumen’s asymmetric location allowed us to generate gradients of nutrient, pH, and cell viability across the model. Consequently, depending on the cell density used, gradients of proliferation and cell viability were generated in the microdevice. Those cells located in the innermost region of the microdevice died, whereas cells located near the lumen remained viable and proliferated. Interestingly, cells located in the middle region remained viable but showed no proliferation, remaining quiescent. The polydimethylsiloxane (PDMS)-based microdevice allowed oxygen and CO_2_ exchange, decoupling the generation of nutrient gradients from potential hypoxia gradients. The microdevice relied on passive diffusion to generate these nutrient gradients instead of using external equipment to provide a more approachable methodology. We used the model to evaluate the effect of cell density and nutrient availability on the sensitivity to metabolic inhibitors targeting tumor metabolism. Hence, we observed that when cultured at low density, breast cancer cells exhibited more resistance to metabolic inhibition. However, when cultured at higher density with compromised nutrient concentrations, cancer cells were more sensitive to metabolic inhibition. Additionally, cancer cells exhibited different sensitivity to metabolic inhibition depending on their location in the model and corresponding access to nutrients.

## 2. Results and Discussion

### 2.1. Cell Density Modulates Necrotic Core Generation

**Figure 1:**
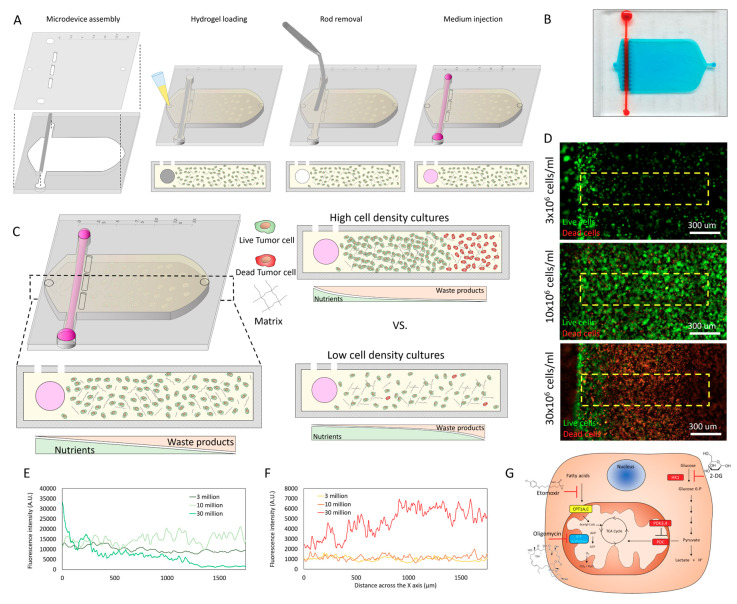
Tumor-on-a-chip platform to evaluate breast cancer metabolic adaptations. (**A**) Schematic description of the microdevice assembly and fabrication. A polydimethylsiloxane (PDMS) rod was placed in the bottom layer of the microdevice layer. Next, the top layer was placed on top, creating a sealed microdevice. A collagen hydrogel mixture containing the cells was pipetted into the central microchamber. After collagen polymerization, the PDMS rod was removed, generating a lumen to perfuse media and nourish the cells. (**B**) Picture of the microdevice. Blue-colored hydrogel was injected in the microchamber, whereas red-colored water was perfused through the lumen for visualization purposes. (**C**) A single lumen in one side of the microdevice allowed us to create gradients of cell viability. A necrotic core could be generated in the microdevice by modifying the cell density used in the microdevice. (**D**) Fluorescence microscopy images showing cell viability in the microdevice. Breast cancer MCF7 cells were cultured in the three-dimensional (3D) hydrogel in the microdevice for 48 h at multiple cell densities (3, 10, and 30 × 10^6^ cells/mL). Live cells are shown in green, whereas dead cells appear in red. (**E**,**F**) Graphs showed the fluorescence intensity profile of live (green line) or dead (red line) across the delimited region shown in panel D (i.e., yellow rectangle). (**G**) Schematic depicting the targets of 2-deoxyglucose (2DG) (i.e., glycolysis inhibitor), etomoxir (i.e., fatty acid oxidation inhibitor), and oligomycin (i.e., adenosine triphosphate (ATP) synthetase inhibitor).

**Figure 2:**
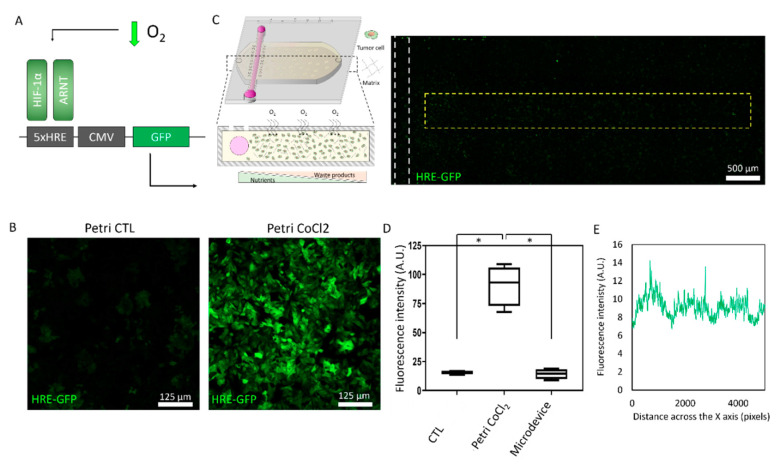
Absence of hypoxic conditions in the microdevice. (**A**) Schematic depicting green fluorescent protein (GFP) expression under hypoxia conditions in the hypoxia-response element GFP (HRE/GFP) plasmid. (**B**) Confocal images showing GFP expression under hypoxia-like conditions. To mimic hypoxia, HRE/GFP MCF7 cells were incubated during 24 h in the presence of 250 µM CoCl_2_, preventing hypoxia-induced factor 1α (HIF-1α) degradation in normoxic conditions and leading to a hypoxia-like phenotype. (**C**) HRE/GFP expression profile across the microdevice. MCF7 cells were cultured at high cell density (i.e., 30 million cells/mL) but no oxygen gradient was observed. (**D**) Bar graph shows HRE/GFP fluorescence intensity in HRE/GFP MCF7 cells cultured in normoxic conditions (CTL), CoCl_2_, and in the microdevice. (**E**) HRE/GFP intensity profile across the delimited region (i.e., yellow rectangle) shown in C. * denotes *p*-value < 0.05.

The tumor-on-a-chip microdevice included a central chamber and a crossing tubular structure. Within the central chamber, breast cancer MCF7 cells were embedded in a 3D collagen hydrogel (Figure 1A). The presence of a lumen in one side of the chamber allowed us to perfuse culture medium to feed the cells, and depending on the cell density, gradients of viability could be established across the chamber (Figure 1B,C). When cultured at low cell density for 48 h (i.e., 3 million cells/mL), the large majority of MCF7 cells remained viable across the hydrogel, showing a sparse cell distribution with larger cell clusters next to the lumen (Figure 1D,F). When we increased the density to 10 million cells/mL, cells occupied most of the chamber while maintaining high cell viability. Conversely, when cultured at 30 million cells/mL, MCF7 cells led to the generation of a necrotic core occupying more than 75% of the microdevice chamber. To disprove the presence of potential gradients of oxygen, we also cultured hypoxia-response element green fluorescent protein (HRE/GFP) MCF7 cells, which were transfected with theGFP protein under a HRE, inducing the expression of GFP under hypoxic conditions (Figure 2). These results demonstrated the effect of cell density on cell viability. Specifically, results suggested that at high cell density, nutrient consumption by cells close to the lumen led to cell death in those cells located far from the lumen. Glucose diffusion through the hydrogel was monitored using a fluorescent glucose analog: 2-(N-(7-Nitrobenz-2-oxa-1,3-diazol-4-yl)Amino)-2-Deoxyglucose (NBDG) (Supporting Figure S1). Next, we set out to explore tumor metabolic vulnerability by blocking different metabolic pathways at different cell densities. We decided to evaluate metabolic vulnerability at 3 million cells/mL (defined as “low density” from now on) and at 15 million cell/mL (“high density”). We decided to study 15 million/mL instead of 30 million cells/mL because of the large necrotic core observed at 30 million cells/mL, which occupied more than 75% of the chamber and, in turn, limited the area of analysis.

### 2.2. Metabolic Vulnerability in Low Cell Density Cultures

**Figure 3:**
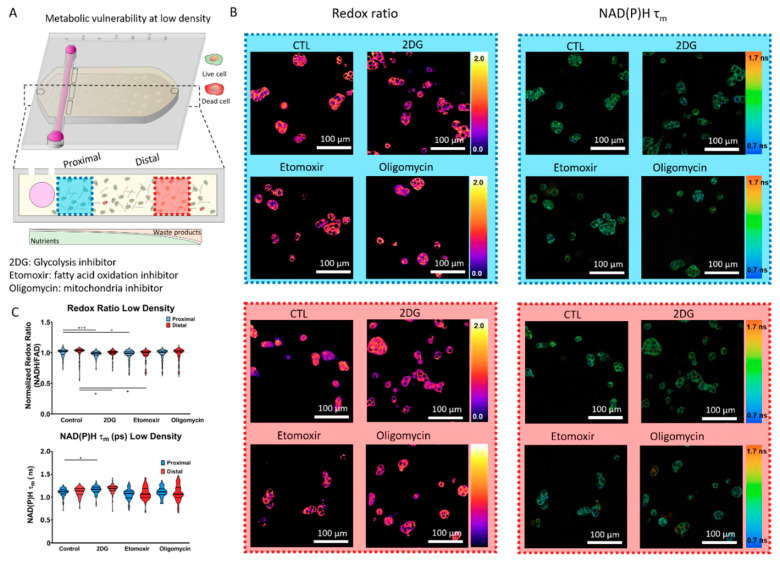
Optical metabolic imaging (OMI) in low cell density cultures. (**A**) Breast cancer MCF7 cells were cultured in the tumor-on-a-chip microdevice at 3 million cells/mL for 24 h in culture medium (CTL), or in the presence of 2-Deoxy-D-glucose (2DG), etomoxir, or oligomycin. After 24 h, cells were imaged using a multi-photon OMI. Nicotinamide adenine (phosphate) dinucleotide (NAD(P)H) and flavin adenine dinucleotide (FAD) autofluorescence were visualized and the ratio between NAD(PH) and FAD intensity was used to calculate the MCF7 cell optical redox ratio. NAD(P)H mean lifetime (τ_m_) was also calculated in nanoseconds (ns) to monitor changes in the MCF7 cell metabolic phenotype. Cell metabolism was evaluated in the proximal (blue square) and distal (red square) area. (**B**) Left panels: Multi-photon OMI shows the optical redox ratio of MCF7 cells located in the proximal and distal area, respectively. Right panels: Multi-photon OMI shows the NAD(P)H τ_m_ of MCF7 cells located in the proximal and distal area, respectively. (**C**) Top graph: Violin plot shows the optical redox ratio normalized to the respective control (proximal or distal area) for each condition. Bottom graph: Violin plot shows the analysis of NAD(P)H τ_m_ in the proximal and distal area. * and *** denote *p*-value < 0.05 and 0.001, respectively.

We cultured MCF7 cells at “low density” (i.e., 3 million cells/mL) in the presence of a glycolysis inhibitor (i.e., 2DG), a fatty acid oxidation inhibitor (i.e., etomoxir), or a TCA cycle inhibitor (i.e., oligomycin) (Figure 3A). After 24 h in culture, we analyzed MCF7 cell metabolic phenotype using optical metabolic imaging (OMI). OMI visualizes nicotinamide adenine (phosphate) dinucleotide (NAD(P)H) and flavin adenine dinucleotide (FAD) auto-fluorescence intensity to calculate the optical cellular redox ratio (i.e., NAD(P)H intensity/FAD intensity). Multi-photon OMI revealed that cells formed similar multicellular clusters next to the lumen (i.e., proximal region) and far from the lumen (i.e., distal region, defined as 10 mm away from the lumen) (Figure 3). These multicellular clusters were also observed in the presence of 2DG, etomoxir, and oligomycin.

Furthermore, quantitative analysis revealed that MCF7 cells located in the proximal and distal area exhibited a similar normalized optical redox ratio, suggesting that at low density (i.e., 3 million cells/mL), nutrient concentration was enough to maintain redox balance. Whereas 2DG or etomoxir led to significant changes in the optical redox ratio, oligomycin did not. These results suggested that despite inhibiting metabolic pathways, MCF7 cells were able to rewire their metabolism to maintain their redox balance. When we analyzed NAD(P)H mean fluorescence lifetime (τ_m_), a physical parameter affected by the intracellular metabolic environment (i.e., proportion of NAD(P)H free vs. bound to enzymes), we again observed moderate to no changes. Altogether, these results indicated that at low density, MCF7 cells tolerated the presence of several metabolic inhibitors while maintaining their redox balance.

**Figure 4:**
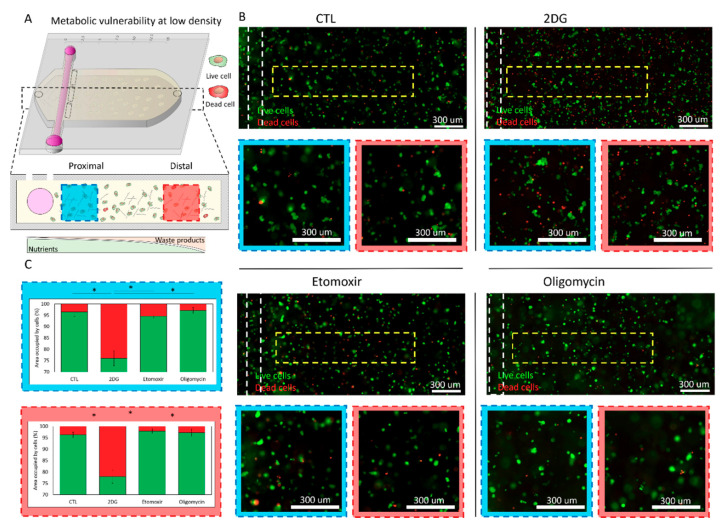
Metabolic vulnerability in low-density cell cultures. (**A**) Breast cancer MCF7 cells were cultured in the tumor-on-a-chip microdevice at 3 million cells/mL for 48 h in culture medium (CTL), 2-Deoxy-D-glucose (2DG), etomoxir, or oligomycin. Cell viability was evaluated using calcein acetoxymethyl (CAM) and propidium iodide (PI), labeling viable and dead cells in green and red, respectively. Cell metabolism was evaluated in the proximal (blue square) and distal (red square) area. (**B**) Fluorescence microscopy images showing cell viability across the collagen hydrogel at low magnification. Panels show cell viability in the proximal (blue square) and distal region (red square). (**C**) Bar graphs analyzing cell viability in the proximal (blue-dotted rectangle) and distal region (red-dotted rectangle) in the presence of 2DG, etomoxir, or oligomycin. Viable percent in green, dead cell percent in red. * denotes *p*-value < 0.05.

Next, we set out to evaluate the potential effect of these metabolic inhibitors on cell viability (Figure 4). After 48 h in culture, we removed the microdevice’s upper layer to homogeneously expose the collagen hydrogel with the MCF7 cells embedded to a cell viability staining. Specifically, we analyzed cell viability using calcein acetoxymethyl (CAM), and propidium iodide (PI), which stained viable and dead cells in green and red, respectively. In the absence of metabolic inhibitors (i.e., control (CTL) conditions), MCF7 cells formed the multicellular clusters observed before and remained viable across the hydrogel, showing similar cell viability in the proximal and distal area (Figure 3B,C). On the other hand, 2DG caused a significant albeit moderate decrease in cell viability (>95% in CTL vs. 75% in 2DG), suggesting that the metabolic perturbation observed at 24 h (Figure 3) ultimately compromised cell viability. Similar to the changes observed in OMI (Figure 3), the decrease in cell viability was observed in both the proximal and the distal area (Figure 4B,C). Finally, the presence of etomoxir or oligomycin did not affect cell viability, suggesting that, at low density, MCF7 cells could bypass the potential effect of metabolic inhibitors targeting fatty acid oxidation (FAO) or ATP synthetase (i.e., oxidative phosphorylation) (Figure 4B,C).

### 2.3. Metabolic Vulnerability in High Cell Density Cultures

**Figure 5:**
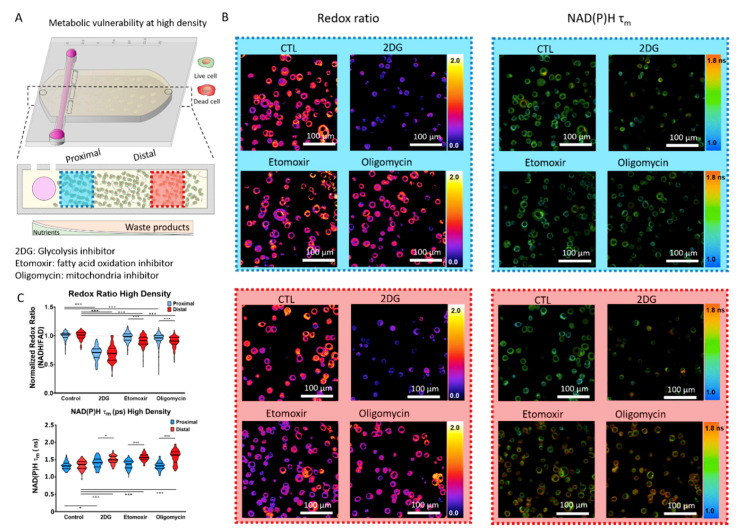
OMI in high cell density cultures. (**A**) Breast cancer MCF7 cells were cultured in the tumor-on-a-chip microdevice at 15 million cells/mL for 24 h in culture medium (CTL), or in the presence of 2-Deoxy-D-glucose (2DG), etomoxir, or oligomycin. After 24 h, cells were imaged using a multi-photon microscopy. NAD(P)H and FAD autofluorescence were visualized and the ratio between NAD(PH) and FAD intensity was used to calculate the MCF7 cell optical redox ratio. NAD(P)H τ_m_ was also calculated to monitor changes in the MCF7 cell metabolic phenotype. Cell metabolism was evaluated in the proximal (blue square) and distal (red square) area. (**B**) Left panels: Multi-photon OMI shows the optical redox ratio for MCF7 cells located in the proximal and distal area, respectively. Right panels: Multi-photon OMI shows the NAD(P)H τ_m_ of MCF7 cells located in the proximal and distal area, respectively. (**C**) Top graph: Violin plot shows the optical redox ratio normalized to the respective control (proximal or distal area) for each condition. Bottom graph: Violin plot shows the analysis of NAD(P)H τ_m_ in the proximal and distal area. * and *** denote *p*-value < 0.05 and 0.001, respectively.

Solid tumors commonly exhibit a central necrotic core caused by rapid nutrient consumption and compromised blood supply. Therefore, tumor cells located far from the vasculature must rewire their metabolism to survive in a nutrient-depleted environment, which in turn may affect their vulnerability to metabolic inhibitors. Thus, we decided to mimic these nutrient-depleted conditions by increasing cell density in the microdevice, thereby raising the metabolic pressure on MCF7 cells. Based on our previous results (Figure 1), we increased cell density to 15 million MCF7 cells/mL (Figure 5A). After 24 h in culture, before the potential formation of the necrotic core, we analyzed MCF7 cell metabolism using OMI. Optical redox ratio analysis demonstrated that, in the absence of metabolic inhibitors, MCF7 cells maintained their redox balance constant in the proximal and distal area. However, the presence of 2DG, etomoxir, or oligomycin led to more severe changes in the high-density condition than in the low-density cultures (Figure 5B,C). At high cell density, 2DG caused a 40% optical redox ratio decrease in MCF7 cells located in the proximal and distal area. This observation suggested that in high-density cultures, MCF7 cells were sensitive to the glycolysis inhibitor regardless of their location inside of the microdevice. Interestingly, etomoxir showed a different trend, leading to a more significant optical redox ratio decrease in the distal area compared with the proximal region. Similarly, oligomycin showed a more prominent effect on cells located in the distal area. These results demonstrated that increased cell density compromised MCF7 cells’ capacity to maintain their redox balance. However, glycolysis, FAO, and the electron transport chain seemed to play a different role in maintaining redox balance when nutrients were compromised. The NAD(P)H τ_m_ analysis revealed an overall increase in NAD(P)H τ_m_ at high cell density compared with low-density cultures (Figure 2 and Figure 4). Additionally, in high-density cultures, MCF7 cells exhibited different metabolic rewiring depending on their location in the microdevice (Figure 5B,C). In this condition, 2DG led to a similar optical redox ratio decrease in the proximal and distal area, but the NAD(P)H τ_m_ analysis showed that the metabolic routes leading to those changes were different between both areas. The increase in NAD(P)H τ_m_ observed in cells located in the distal area in the presence of 2DG suggested a higher ratio of NAD(P)H bound to enzymes (e.g., NAD(P)H consumption in the mitochondria for ATP generation).

**Figure 6:**
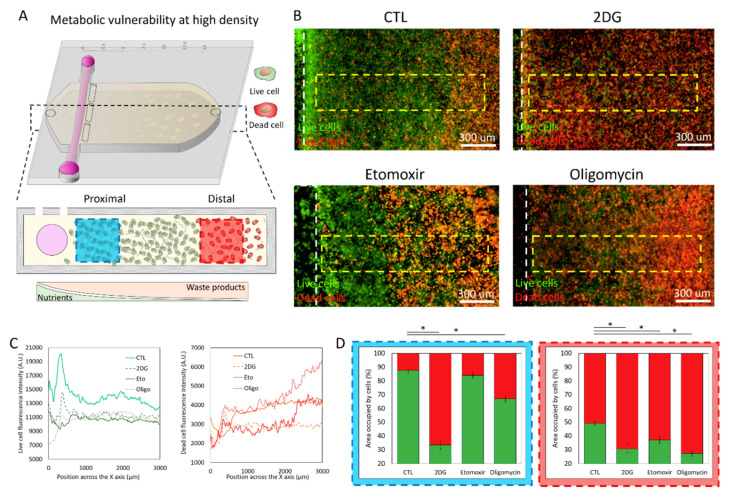
Metabolic vulnerability in high-density cell cultures. (**A**) Breast cancer MCF7 cells were cultured in the tumor-on-a-chip microdevice at 15 million cells/mL for 48 h in culture medium (CTL), 2-Deoxy-D-glucose (2DG), etomoxir, or oligomycin. Cell viability was evaluated using CAM and PI, labelling viable and dead cells in green and red, respectively. (**B**) Fluorescence microscopy images showing cell viability across the collagen hydrogel at low magnification in the presence of culture medium (CTL), 2DG, etomoxir, or oligomycin. (**C**) Graphs show the fluorescence intensity profile of live (green) and dead (red) cells across the delimited region highlighted with a yellow rectangle in (**B**). (**D**) Bar graphs analyzing cell viability in the proximal (blue-dotted rectangle) and distal region (red-dotted rectangle) in the presence of 2DG, etomoxir, or oligomycin. Viable percent in green, dead cell percent in red. * denotes *p*-value < 0.05.

Overall, these results demonstrated how changes in cell density affect cell metabolism in the microdevice. Thus, we decided to evaluate the potential effect of metabolic inhibition on cell viability (Figure 6A). After 48 h in culture, the cell viability images demonstrated an emerging necrotic core at the furthest region of the microdevice (Figure 6B,C), suggesting a more intense nutrient depletion than low-density cultures (Figure 4B and Figure 6B). In high-density cultures, 2DG caused a severe decrease in cell viability in the proximal (87% vs. 33%) and a moderate decrease in the distal area (50% vs. 30%). Our OMI analysis revealed that 2DG was affecting cells located in the proximal and distal areas (Figure 5B,C), suggesting that the metabolic changes observed at 24 h preceded the cellular necrosis visualized at 48 h. The addition of etomoxir only decreased cell viability in the distal region (50% vs. 36%). Interestingly, oligomycin caused a significant albeit moderate decrease in viability in the proximal area (87% vs. 66%), whereas the effects on the distal area were more severe (87% vs. 27%). In this context, both etomoxir and oligomycin increased the size of the necrotic core, destroying cells far from the lumen that were alive in the control experiments. This observation suggested that those live cells located far from the lumen relied on FAO and ATP synthetase to survive in high-density cultures. Altogether, these results demonstrated that MCF7 cells were more vulnerable to metabolic inhibitors at high-density than low-density cultures (Figure 4 and Figure 6), highlighting the critical role of the metabolic and nutrient environment on drug response.

### 2.4. Metabolic Plasticity

**Figure 7:**
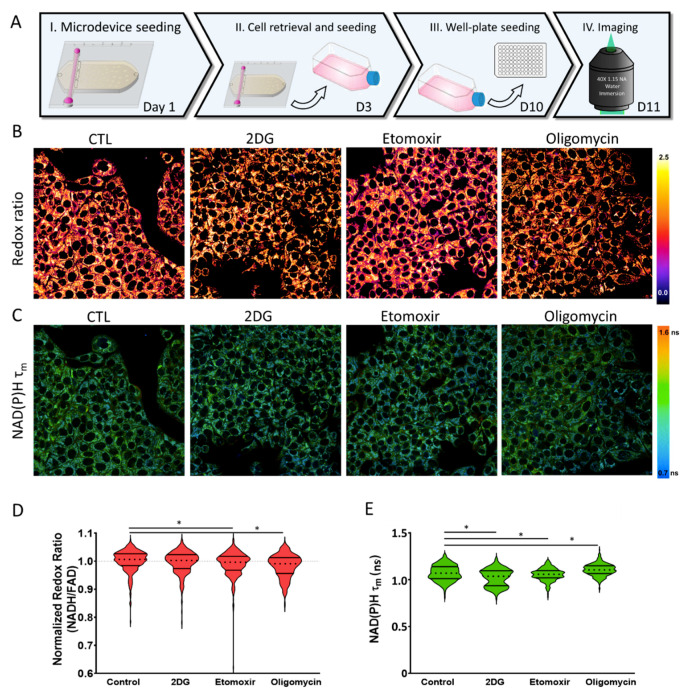
Metabolic analysis of recovered cells. (**A**) 15 million GFP-MCF7 cells/mL were cultured in the tumor-on-a-chip microdevice for 48 h in the presence of 2DG, etomoxir, or oligomycin. Next, cells were retrieved from the microdevice and cultured in a traditional Petri dish for 7 days in culture medium. 5000 MCF7 cells were reseeded on 96-well-plates and OMI was monitored after 24 h. (**B**) OMI multi-photon images showing the normalized optical redox ratio in the presence of culture medium (CTL), 2DG, etomoxir, or oligomycin. Images were normalized to the control (CTL). (**C**) OMI multi-photon images showing NAD(P)H τ_m_ in the presence of culture medium (CTL), 2DG, etomoxir, or oligomycin. (**D**,**E**) Violin plots of the optical redox ratio normalized to the control condition, and NAD(P)H τ_m_ across recovered cell conditions. * denotes *p*-value < 0.05.

The OMI and cell viability analyses revealed that nutrient depletion in high cell density cultures led to changes in metabolic vulnerability. Next, we set out to evaluate whether these changes in cell metabolism were transient or permanent. Thus, we cultured MCF7 cells at high cell density in the microdevice for 48 h and then degraded the collagen matrix, which released the cells from the hydrogel to reseed them in traditional cell culture flasks. We cultured the recovered cells for 7 days and then seeded them in glass-bottom dishes to analyze cell metabolism via OMI (Figure 7). OMI analysis demonstrated that the changes caused by 2DG in the microdevice disappeared in the recovered cells, showing a similar optical redox ratio compared with the control and a minor change in NAD(P)H τ_m_. Recovered cells that were exposed to etomoxir and oligomycin showed only minor changes in optical redox ratio as well as NAD(P)H τ_m_ compared with the more intense changes observed in the high-density cultures (Figure 5 and Figure 7). These observations demonstrated that multiple metabolic adaptations observed in the microdevice in the presence of metabolic inhibitors were transient, further highlighting the importance of the metabolic environment when targeting tumor metabolism.

**Figure 8:**
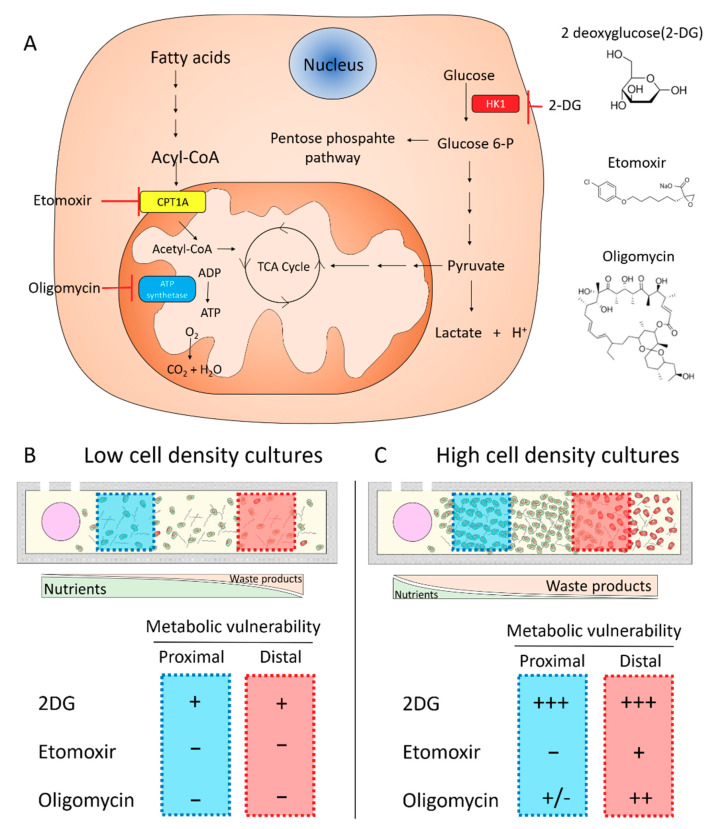
Effect of culture density on tumor metabolic vulnerability. (**A**) Schematic depicting the main targets of the metabolic inhibitors tested. HK: hexokinase, CPT: carnitine palmitoyltransferase, TCA: tricarboxylic cycle. (**B**) When cultured at “low density” (i.e., 3 million cells/mL), MCF7 cells exhibited low sensitivity to metabolic inhibitors. Glycolysis inhibitor 2DG showed a moderate effect on cell viability in the proximal and distal region, whereas etomoxir (i.e., fatty acid oxidation inhibitor) and oligomycin (i.e., ATP synthetase inhibitor) demonstrated no effect. (**C**) In high-density cultures, MCF7 cells showed greater responses to the same metabolic inhibitors. 2DG led to a significant change in optical redox ratio, NAD(P)H τ_m_, and cell viability. Oligomycin showed a moderate effect, most affecting those cells located in the distal area. Etomoxir led to minor changes in the distal area.

Solid tumors present an evolving microenvironment where rapid nutrient consumption and compromised blood supply leads to nutrient and waste product gradients. Consequently, those tumor cells growing in nutrient-depleted environments activate multiple adaptive responses, including changes in cell metabolism. Thus, effective therapies targeting tumor metabolism must consider tumor heterogeneity. However, most metabolic inhibitors are still tested in traditional Petri dishes, which struggle to capture this complex and heterogeneous environment. In this manuscript, we observed the profound effect of cell density and nutrient availability on cell metabolism and metabolic vulnerability. When cultured at low density, MCF7 cells showed low sensitivity to 2DG, whereas etomoxir and oligomycin had no significant effect. 2DG, etomoxir, and oligomycin target glycolysis (and the pentose phosphate pathway), FAO, and oxidative phosphorylation, respectively. These metabolic pathways are involved in multiple different metabolic routes, including ATP production and macromolecule synthesis, but they also share some overlapping functions (e.g., glycolysis, FAO, and oxidative phosphorylation can generate energy for the cell in the form of ATP) (Figure 8A). Thus, we hypothesize that at low cell density, MCF7 cells had enough nutrients available to survive the metabolic blockade caused by 2DG, etomoxir, and oligomycin (Figure 8B). Conversely, in high cell density cultures, MCF7 cells were significantly more sensitive to these metabolic inhibitors (Figure 8C). In high-density cultures, hexokinase blocking (i.e., 2DG) led to multiple changes in cell metabolism and a severe reduction in cell viability. These results suggested that in a nutrient-depleted environment (i.e., high-density cultures), MCF7 cells were more dependent on glucose metabolism (i.e., glycolysis, pentose phosphate pathway) to survive. Similarly, in high-density cultures, inhibition of FAO (etomoxir) or the mitochondrial ATP synthetase (oligomycin) had a larger effect in cell metabolism and cell viability than in low-density cultures. In this study, we limited our analysis to specific time-points, but future studies suing this methodology could analyze in real time the pharmacodynamics of metabolic inhibitors [23,24,25,26]. These studies could evaluate the capacity of cells located in different positions of the chamber adapted to metabolic inhibition over time. Adaptative or compensatory mechanisms leading to upregulation or downregulation of specific metabolic pathways could enable cancer cells to endure metabolic inhibition or provide clinicians with other targetable enzymes.

## 3. Conclusions

Tumor cells present considerable metabolic differences compared with normal cells, which may offer new potential therapeutic opportunities. In a previous study, we used OMI to monitor metabolic changes in mammary fibroblasts, normal, and tumor mammary cells. The analysis demonstrated that these cells exhibited different redox ratio and NAD(P)H lifetime. Additionally, multiple studies have explored the molecular changes driving these metabolic adaptations [12,24,27,28]. However, solid tumors are heterogeneous systems where multiple metabolic phenotypes co-exist and evolve across the tumor mass. Our platform provides a versatile yet simple tool to generate gradients of nutrients, pH, and viability across a 3D hydrogel. In this manuscript, we used this technology to monitor changes in cell metabolism and cell viability at different cell concentrations. We observed that the fluorescent glucose analog (MW 342 g/mol) rapidly penetrated through the hydrogel, suggesting that small nutrients such as amino acids or glucose could nourish cells located far from the lumen. Other studies could use this technology to explore whether hydrogel structure could hinder the penetration of larger nutrients such as proteins or lipid vesicles. More importantly, we used the microdevice to study how tumor vulnerability to metabolic blockade depends on the surrounding environment. We observed that in the presence of a nutrient-enriched environment (i.e., low cell density cultures), tumor cells were able to survive in the presence of metabolic inhibitors targeting glycolysis, FAO, or oxidative phosphorylation. However, when nutrient supply was compromised (i.e., high-density cultures), tumor cells were more vulnerable to these inhibitors. Enabled by this technology, future studies could include multiple cell types (e.g., cancer-associated fibroblasts, cancer-associated macrophages) [25], evaluate the potential of new metabolic inhibitors, or explore combinatorial therapy to develop more efficient treatments against solid tumors.

## 4. Materials and Methods

### 4.1. Tumor-on-a-Chip Microdevice Fabrication

The tumor-on-a-chip microdevice was fabricated following the protocol described in [18]. Briefly, the design was generated in illustrator to generate a photomask. Next, a SU-8 (i.e., epoxy-based negative photoresist)-based template was fabricated using UV lithography. The final tumor-on-a-chip microdevice was fabricated by pouring PDMS on top of the SU-8 templates, followed by incubation at 80 °C for 4 h. The bottom and top layers of the microdevice were assembled together and bonded to a 60 mm-diameter glass-bottom Petri dish using oxygen plasma. This approach yielded a non-permanent bond between the two PDMS layers and a permanent bond between the PDMS and the glass-bottom Petri dish. The top layer of the microdevice could be removed to expose the collagen hydrogel without disturbing it to retrieve the cell for downstream analysis. The lumen structure was generated using a 340 µm-diameter PDMS rod inserted through the microchamber. Tumor-on-a-chip microdevices were sterilized, exposing them to UV light for 15 min

### 4.2. Cell Culture

Breast cancer MCF7 cells were cultured in DMEM (Thermo Fisher, Madison, WI, USA, 10566016) supplemented with 10% FBS (Thermo Fisher, Madison, WI, USA, 26140079). Cells were used form passage #3 to #15. To culture MCF7 cells in the tumor-on-a-chip microdevice, MCF7 cells were trypsinized and resuspended at the desired density (i.e., 60, 20, 6 million cells/mL). Then, a 2.0 mg/mL collagen hydrogel mixture containing 30, 10, or 3 million MCF7 cells/mL was prepared as follows: 38.9 H_2_O, 10 μL of 10 × PBS, 2.45 μL of 1 M NaOH, 48.8 μL of 8.43 mg/mL rat tail collagen type I, and 100 μL of MCF7 cell suspension. Then, the collagen hydrogel was injected into the microdevice chamber and polymerized at room temperature for 20 min. The PDMS rod was removed using sterilized tweezers after collagen polymerization, thus yielding a lumen structure through the collagen hydrogel to perfuse media and feed the cells inside the tumor-on-a-chip microdevice. Finally, 5 mL of cell culture media was then pipetted to the Petri dish before placing the microdevices in an incubator at 37 °C with 5% CO_2_.

### 4.3. HRE-GFP Transfection

MCF7 cells were transfected with a HRE/GFP plasmid, which encoded a GFP controlled by 5 hypoxia-response elements (HRE). Plasmid transfection led to GFP expression under hypoxic conditions. Briefly, HRE/GFP was a gift from Martin Brown and Thomas Foster (Addgene, plasmid # 46926). The plasmid was purified using the QIAprep Spin Miniprep Kit (Quiagen, Madison, WI, USA, 27104) and transfected in MCF7 using Xfect transfection reagent (Takara, Middleotn, WI, USA, 631317). Briefly, 5 µg of the plasmid was combined with 1.5 µL of Xfect transfection reagent in 88.5 µL of Xfect transfection buffer and added to the cells for 4 h. After 24 h, media was then supplemented with 750 ng/mL G418 to destroy non-transfected cells.

### 4.4. Metabolic Inhibitors

2DG, etomoxir, and oligomycin we purchased from Sigma-Aldrich and dissolved following the supplier instructions. To test MCF7 cell vulnerability to these metabolic inhibitors, 2DG, etomoxir, and oligomycin were diluted in culture medium at 10 mM, 10 µM, and 1 µM, respectively. 10 µM 2DG has been reported to reduce the amount of available ATP, reduce glycolysis, and induce metabolic stress [29,30]. 1 µM Oligomycin induces metabolic stress and lower the levels of oxidative respiration in MCF7 cells [26,27]. Finally, increasing the dose beyond 10 µM etomoxir did not increase the inhibition of fatty acid oxidation in MCF7 cells, and 10 µM etomoxir completely blocked CPT1A binding activity in fatty acid oxidation [28]. 2-(N-(7-Nitrobenz-2-oxa-1,3-diazol-4-yl)Amino)-2-Deoxyglucose (NBDG) was purchased from Thermo Fisher and dissolved at 20 mM in water. NBDG was used at 200 µM in PBS to analyze glucose diffusion through the collagen hydrogel. To ensure the inhibitor concentration remained constant across the microdevice, these compounds were added in the hydrogel mixture as well as in the culture medium pipetted on top of the microdevice after collagen polymerization.

### 4.5. Cell Viability

Tumor cell viability was evaluated using calcein acetomethyl ester (CAM) (Thermo Fisher, Madison, WI, USA, C3100MP) and propidium iodide (PI) (Thermo Fisher, Madison, WI, USA, P1304MP), which label viable and dead cells in green and red, respectively. In order to ensure a homogenous staining, the upper layer of the microdevice was removed to expose the collagen hydrogel and then 5 mg/mL CAM and 2 mg/mL PI stock solutions were diluted at 1:1000 and 1:500 respectively, in PBS and added on top of the hydrogel. After 15 min, cell viability was evaluated using a Leica SP8 3X stimulated emission depletion microscopy (STED) super-resolution confocal microscope.

### 4.6. Hydrogel Degradation and Cell Recovery

To retrieve viable MCF7 cells from the microdevice, the upper half of the microdevice was removed to expose the collagen hydrogel [31]. The hydrogel was then transferred to an Eppendorf tube containing 6 mg/mL type I collagenase for 2 min at 37 °C to degrade the collagen fibers and release the cells. Next, the cells were centrifuged at 400 g for 3 min, washed twice in culture media to remove to remove the excess of collagenase, and reseeded in a traditional 25 cm^2^ culture flask.

### 4.7. Optical Metabolic Imaging

A custom-built inverted multiphoton microscope (Bruker Fluorescence Microscopy, Middleton, WI, USA), was used to acquire fluorescence intensity and lifetime images. The equipment consists of an ultrafast laser (Spectra Physics, Insight DSDual), an inverted microscope (Nikon, Eclipse Ti), and a 40× water immersion (1.15 NA, Nikon) objective. Next, NAD(P)H and FAD images were obtained for the same field of view. FAD fluorescence was isolated using an emission bandpass filter of 550/100 nm and excitation wavelength of 890 nm. NAD(P)H fluorescence was isolated using an emission bandpass filter of 440/80 nm and an excitation wavelength of 750 nm. Subsequently, fluorescence lifetime images were collected using time-correlated single-photon counting electronics (SPC-150, Becker and Hickl, Brookline, MA, USA) and a GaAsP photomultiplier tube (H7422P-40, Hamamatsu, Bridgewater, NJ, USA). 512-pixel images were obtained using a pixel dwell time of 4.8 µs over 60 s total integration time. To guarantee adequate photon observations for lifetime decay fits and no photobleaching, the photon count rates were maintained at 1–2 × 10^5^ photons/s. The instrument response function was calculated from the second harmonic generation of urea crystals excited at 900 nm, and the full width at half maximum (FWHM) was measured to be 244 ps. A Fluoresbrite YG microsphere (Polysciences Inc., Warrington, PA, USA) was imaged as a daily standard for fluorescence lifetime. The lifetime decay curves for the YG microsphere standard were fit to a single exponential decay and the fluorescence lifetime was measured to be 2.1 ns (*n* = 7), which is consistent with published values. Collagen second harmonic generation images were generated using an excitation wavelength of 790 nm and an emission bandpass filter of 440/80 nm.

### 4.8. Image and Analysis

Optical redox ratio values for all conditions were normalized to the control condition for the same position (proximal or distal). NAD(P)H and FAD intensity and lifetime images were analyzed using SPCImage software (Becker &Hickl, Berlin, Germany) as described previously [29,30]. The fluorescence lifetime decay curve was deconvolved with the instrument response function and fit to a two-component exponential decay model at each pixel, I(t) = α1 ∗ e(−t/τ1) + α2 ∗ e(−t/τ2) + C, where I(t) represents the fluorescence intensity at time t after the laser excitation pulse, α accounts for the fractional contribution from each component, C represents the background light, and τ is the fluorescence lifetime of each component. Since both NAD(P)H and FAD can exist in two conformational states, bound or unbound to enzymes, a two-component model was used. The short and long lifetime components reflect the bound and unbound conformations respectively, for FAD. While the opposite is true for NAD(P)H, the short and long lifetime components correspond with the unbound and bound conformations, respectively. The mean lifetime (τm) was calculated using τm = α1τ1 + α2τ2, for both NAD(P)H and FAD. The optical redox ratio was determined from the NAD(P)H and FAD intensity images. For each pixel, the intensity of NAD(P)H was then divided by the intensity of FAD. Using Cell Profiler, an automated cell segmentation pipeline was created [29]. This system identified pixels belonging to nuclear regions by using a customized threshold code. Cells were recognized by propagating out from the nuclei within the image. To refine the propagation and to prevent it from continuing into background pixels, an Otsu Global threshold was used. The cell cytoplasm was defined as the cell borders minus the nucleus. Values for NAD(P)H τm, FAD τm, NAD(P)H intensity, FAD intensity, and the optical redox ratio (NAD(P)H/FAD intensity) were averaged for all pixels within each cell cytoplasm. At least 100 cells per sample were analyzed. Cell viability microscopy images were analyzed using FIJI (https://imagej.net/Fiji/Downloads).

### 4.9. Statistical Analysis

All experiments were repeated at least 3 independent times. The normal distribution assumption for statistical tests was confirmed by the Kolmogorov–Smirnov test. Statistical significance was set at *p* < 0.05. For nonparametric comparisons, a Kruskal–Wallis test was performed followed by the Mann–Whitney U test. Analysis was performed in GraphPah Prism 8 (https://www.graphpad.com/scientific-software/prism/).

## Supplementary Materials

The following are available online at https://www.mdpi.com/1422-0067/21/23/9075/s1, Figure S1: Glucose diffusion. (A) Second harmonic generation (SHG) images showed the effect of collagen density and polymerization temperature on collagen fiber organization. Collagen hydrogels polymerized at 37 °C showed shorter fibers and smaller pore size compared hydrogels polymerized at 20 °C. Increasing collagen concentration increased fiber density. (B) 200 µM NBDG (i.e., fluorescent glucose analog) was perfused through the lumen and diffusion was analyzed after 10 min. NBDG rapidly penetrate through 1.5 mg/mL (polymerized at room temperature) and 6 mg/mL collagen hydrogels (polymerized at 37 °C).
