# Supplementary material for: Microfluidic Tumor-on-a-Chip Model to Study Tumor Metabolic Vulnerability
Source: Int J Mol Sci. 2020 Nov 28;21(23):9075. doi: 10.3390/ijms21239075 (PMC7730115; doi:10.3390/ijms21239075)
Supplement: Supplementary file 1 [file ijms-21-09075-s001.pdf]

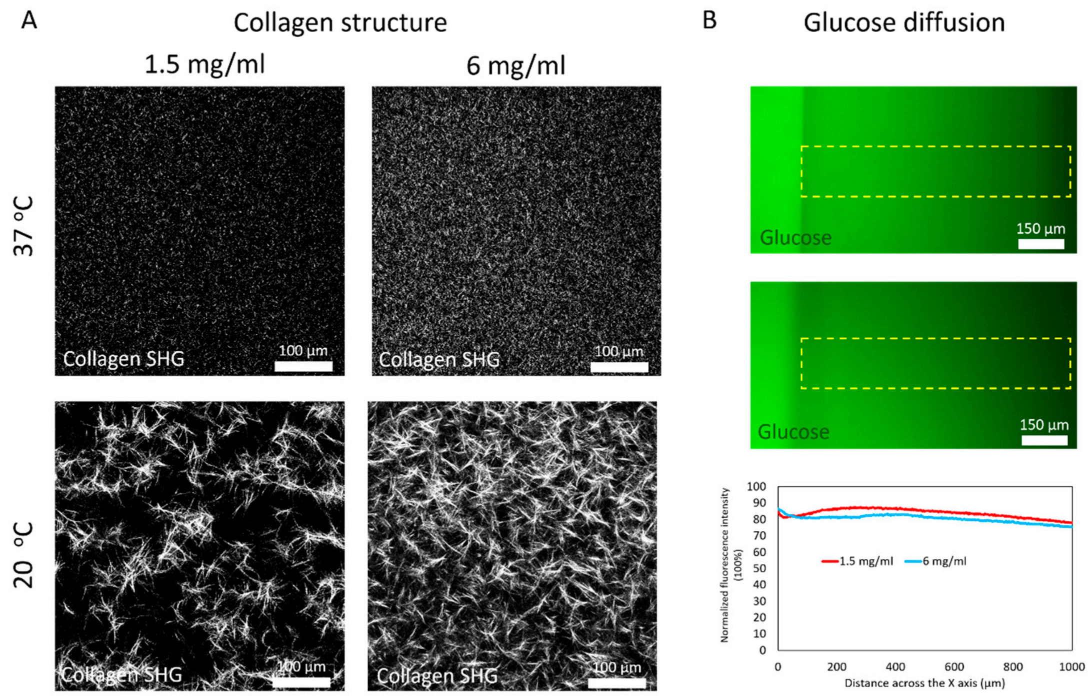

Supporting Figure S1. Glucose diffusion. (**A**) Second harmonic generation (SHG) images showed the effect of collagen density and polymerization temperature on collagen fiber organization. Collagen hydrogels polymerized at 37 °C showed shorter fibers and smaller pore size compared to hydrogels polymerized at 20 °C. Increasing collagen concentration increased fiber density. (**B**) 200 µM NBDG (i.e., fluorescent glucose analog) was perfused through the lumen and diffusion was analyzed after 10 min. NBDG rapidly penetrate through 1.5 mg/mL (polymerized at room temperature) and 6 mg/mL collagen hydrogels (polymerized at 37 °C).
